# Cryptogenic Organizing Pneumonia Is Associated With Increased Mortality Risk in Hospitalizations for Systemic Lupus Erythematosus (SLE): A National Inpatient Sample Analysis

**DOI:** 10.7759/cureus.69901

**Published:** 2024-09-22

**Authors:** Fidelis E Uwumiro, Arji Emmanuel, Christian Offiah, Nnaedozie Umeani, Adaobi Ozigbo, Courage Idahor, Daniel Udegbe, Sobechukwu Chiegboka, Ihunanya Kanu, Magaret Utibe, Marvis Enyi, Samuel C Ayogu, Adaeze B Eze

**Affiliations:** 1 Internal Medicine, Prime Healthcare-Southern Regional Georgia, Riverdale, USA; 2 Internal Medicine, Ulster University Hospital, Belfast, GBR; 3 Internal Medicine, Chukwuemeka Odumegwu Ojukwu University College of Medical Sciences, Uli, NGA; 4 General Practice, College of Medicine, University of Lagos, Lagos, NGA; 5 Internal Medicine, University at Albany - State University of New York, New York, USA; 6 Emergency Medicine, Barking, Harvering and Redbridge Foundation Trust, London, GBR; 7 Psychiatry, Godfrey Okoye University Teaching Hospital, Enugu, NGA; 8 Internal Medicine, Vinnytsia National Medical University, Vinnytsia Oblast, UKR; 9 Internal Medicine, Jackson State University, Jackson, USA; 10 Emergency Medicine, Evercare Hospital Lekki, Choba, NGA; 11 Internal Medicine, Imo State University Teaching Hospital, Owerri, NGA; 12 Internal Medicine, St Hugh's Hospital, Grimsby, GBR; 13 Internal Medicine, Regions Stroke and Neuroscience Hospital, Owerri, NGA

**Keywords:** bronchiolitis obliterans organizing pneumonia, idiopathic cryptogenic organizing pneumonia, lung involvement in sle, secondary organizing pneumonia, systemic lupus erythematosus

## Abstract

Background

This study analyzed the incidence, characteristics, and mortality risk associated with cryptogenic organizing pneumonia (COP) among hospitalizations for systemic lupus erythematosus (SLE) with lung involvement.

Methods

Adult hospitalizations from the 2016-2020 nationwide inpatient sample were analyzed using relevant International Classification of Diseases (ICD)-10 codes for SLE with lung involvement (M32.13) and COP (J84.116). We compared baseline characteristics of individuals with SLE and COP to those of other lung involvements using Chi-square tests for categorical variables and the Wilcoxon rank sum test for continuous variables. A Cox proportional hazards model was used to assess the risk of developing COP in the pooled cohort of SLE patients. The impact of COP on SLE mortality was assessed using multivariate logistic regression adjusting for illness severity, baseline risk of mortality at admission, and patient- and hospital-level covariates.

Results

Of 40,356 admissions for SLE, 3,175 (7.9%) were due to lung involvement, with COP identified in 570 cases (17.9%). Compared with other lung involvement in SLE, individuals with COP were significantly older (mean age: 65 vs. 44.3 years; p<0.001), mostly female (515; 90.4% vs. 2,305 males; 88.5%; p=0.572), had a greater baseline risk of mortality [diagnosis-related groups (DRG) major or extreme likelihood of dying: 360; 63.1% vs. 1,133; 43.5%; p<0.001], and had a higher prevalence of peripheral vascular disease (25; 4.4% vs. 39; 1.5%; p<0.001), and lower prevalence of lymphocytopenia (45; 7.9% vs. 359; 13.8%; p=0.001), and hypothyroidism (44; 7.8% vs. 357; 13.7%; p=0.001). Predictors of COP included female sex [adjusted hazard ratio (AHR): 1.46; 95% confidence interval (CI): 1.12-2.96; p=0.022]; hospitalizations occurring in the third quarter of the year (AHR: 1.37; 95% CI: 1.05-2.23; p=0.038); hospital stays of six days or longer (AHR: 1.71; 95% CI: 1.06-2.77; p=0.029); undergoing five or more procedures during the same hospitalization (AHR: 1.56; 95% CI: 1.26-3.56; p=0.041); coexisting lymphocytopenia (AHR: 1.92; 95% CI: 1.16-3.19; p=0.011); need for mechanical ventilation (AHR: 1.60; 95% CI: 1.48-3.93; p=0.049), presence of another autoimmune disorder (AHR: 1.37; 95% CI: 1.15-4.29; p=0.040), and being hospitalized at private, investor-owned hospitals (AHR: 2.62; 95% CI: 1.03-6.64; p=0.043). Mortality in SLE with lung involvement was correlated with age ≥ 60 years [hazard ratio (HR) (95% CI) 1.16 (1.05-1.56); p=0.012], coexisting lupus nephritis [HR (95% CI), 2.44 (2.04-3.49); p=0.031], cancer [HR (95% CI), 3.49 (2.19-5.79); p<0.001], liver disease [HR (95% CI), 9.82 (4.79-12.57); p<0.001]; immune deficiency [HR (95% CI), 2.22 (2.02-3.11); p=0.031], hypothyroidism [HR (95% CI), 4.67 (1.47-7.75); p=0.009], and high blood pressure [HR (95% CI), 3.15 (2.83-4.51); p<0.001]. In the multivariable analysis, COP remained significantly associated with an increased risk of mortality [AHR (95% CI), 1.43 (1.16-2.74); p=0.031]. The incidence of COP did not significantly impact hospitalization costs ($US 94,772 ± 14,759 vs. 95,982 ± 32,625; p=0.954) or length of stay (mean length of hospital stay: 8.3 vs.6.8 days; p=0.147).

Conclusion

Cryptogenic organizing pneumonia was associated with 1% of all hospitalizations for SLE and 18% of cases involving lung complications in SLE. The presence of COP significantly increased the risk of mortality in SLE patients with lung involvement.

## Introduction

Systemic lupus erythematosus (SLE) is characterized by its heterogeneity in clinical manifestations and affects multiple organs, including the lungs. Lung involvement in SLE is a significant contributor to morbidity and mortality, and it presents in various forms, ranging from pleuritis to various interstitial lung diseases [1,2]. Cryptogenic organizing pneumonia (COP) is a distinct clinicopathologic entity characterized by the presence of intra-alveolar buds of granulation tissue, which is usually associated with organizing pneumonia due to an unspecified causative factor [3-6]. The presenting symptoms are often nonspecific and may include dry cough, shortness of breath, wheezing, weight loss, malaise, chest pain, and hemoptysis [3-6]. Although COPs have been extensively studied in various contexts, their specific frequency in SLE and impact on patient outcomes remain poorly explored.

There is a paucity of data on the incidence of COP and its effects on the outcomes of hospitalizations for lung involvement in SLE. This study assessed the incidence and prognostic significance of COP in patients with SLE hospitalized due to lung complications. By analyzing a nationwide cohort of SLE patients with confirmed lung involvement, this study aims to shed light on the clinical features, patient demographics, risk factors for COP, and associated mortality rates of SLE patients with COP. 

## Materials and methods

Study population and data source

Data for all patients admitted between January 2016 and December 2020 across U.S. hospitals with at least one SLE diagnosis were retrieved from the Nationwide Inpatient Sample Database (NIS). The NIS is part of a collection of databases and software tools developed for the Healthcare Cost and Utilization Project (HCUP) [7]. The NIS is the largest publicly available all-payer inpatient healthcare database designed to produce U.S. regional and national estimates of inpatient utilization, access, costs, quality of care, and outcomes. Unweighted, it contains data from approximately seven million hospital stays per year. Weighted, it estimates approximately 35 million nationwide hospitalizations every year. Developed through a federal-state-industry partnership sponsored by the Agency for Healthcare Research and Quality (AHRQ), HCUP data inform decision-making at the national, state, and community levels. The NIS encompasses a broad range of data elements, combining clinical and resource-use information akin to those found in hospital discharge abstracts while implementing stringent privacy protections for patients, physicians, and hospitals. The database incorporates both clinical and nonclinical details for each hospital stay, including diagnostic and procedural codes from the International Classification of Diseases, Ninth Revision, Clinical Modification (ICD-9-CM) for entries before October 1, 2015, and from the Tenth Revision (ICD-10-CM/PCS) thereafter [8,9]. It captures patient demographics such as sex, age, race, and median household income by ZIP code, alongside hospital characteristics such as location, teaching status, region, bed size, and ownership. Other vital information includes the expected primary payer (Medicare, Medicaid, and private insurance or self-pay), total charges associated with the stay, type of hospital discharge, length of hospital stay, in-hospital complications, and metrics on baseline illness severity, risk of mortality, and comorbidity burden at the time of admission [10]. The comprehensive nature of the NIS database makes it an invaluable resource for analyzing healthcare trends, outcomes, and policy impacts.

In the NIS, patient encounters are detailed with both primary and secondary diagnoses, thereby capturing the complexity of hospital stays. The primary diagnosis is the main condition treated or investigated during the hospital admission, essentially the reason for the hospital stay. Secondary diagnoses, conversely, encompass coexisting conditions or complications that arise during the hospitalization, which may affect patient management and outcomes. The NIS records one primary diagnosis and up to 39 secondary diagnoses for each hospitalization. Of note, the United States adopted the ICD-10-CM/Procedure Coding System (PCS) on October 1, 2015. Accordingly, for the current study, diagnoses were coded according to the ICD-10-CM/PCS (available at https://icd.who.int/browse10/2019/en). The reliability and validity of NIS data have been assessed elsewhere [11,12].

To identify the SLE population within the NIS database, we initially extracted records of patients who had at least one instance of the ICD-10 code M32 (indicative of 'systemic lupus erythematosus') listed as either a primary or secondary diagnosis. We excluded any hospitalization for juvenile SLE (patients ≤ 16 years old). Within this selected group, we identified hospitalizations where the ICD-10 code M32.13, denoting 'lung involvement in SLE (LISLE),' was recorded as a primary or secondary diagnosis. Cryptogenic organizing pneumonia (COP) was identified using the ICD-10 diagnosis code J84.116, which includes idiopathic bronchiolitis obliterans organizing pneumonia and organizing pneumonia not otherwise specified. We also used specific ICD-10 codes to identify lupus-associated autoimmune diseases (AIDs), such as Sjögren’s syndrome (M35.0), mixed connective tissue disorder (M35.1), Raynaud’s syndrome (I73.0), systemic sclerosis (M34.X), calcinosis, Raynaud's phenomenon, esophageal dysmotility, sclerodactyly, and telangiectasia (CREST) syndrome (M34.1), antiphospholipid syndrome (D68.61), autoimmune thyroiditis (E063), autoimmune hemolytic anemia (D59), idiopathic thrombocytopenia purpura (D69.3), celiac disease (K90.0), rheumatoid arthritis (M05.X), and inflammatory myopathy (M33.X).

The outcomes of interest in the study were the incidence, characteristics, and mortality risk associated with COP in hospitalizations for SLE. The index study follows the methodological design checklist proposed by Khera et al. (2017) [13] to mitigate typical design errors and enhance the validity and generalizability of our findings.

Statistical analysis

All analyses were performed and findings are presented using the weighted sample, incorporating adjustments for clustering (HOSP_NIS), weighting (DISCWT), and stratification (NIS_STRATUM) within the NIS to ensure that the results accurately reflect the broader U.S. population.

First, we compared sociodemographic characteristics of SLE hospitalizations complicated by COP to other lung involvement in SLE (LISLE). The normality of the distribution of continuous data was assessed using the Shapiro-Wilk test. Categorical variables were analyzed and presented as absolute counts with percentages and evaluated using χ² tests and Student's t‐tests, as appropriate. Continuous variables were described using means and standard deviations if normally distributed, whereas medians and interquartile ranges were applied to variables with a nonnormal distribution. For continuous variables not adhering to a normal distribution, Wilcoxon signed-rank tests were used for analysis.

Subsequently, hazard ratios (HR) for both univariable and multivariable analyses, assessing the risk of developing COP, risk of mortality from SLE, and impact of COP during hospital stays, were determined using Cox proportional hazard models while adjusting for patient- and hospital-level covariates, as well as illness severity and baseline risk of mortality at time of admission. The resulting HRs were reported alongside their 95% confidence intervals (CI) as HRs (95% CI). For the multivariable Cox proportional hazard analysis, variables that showed a significance level of <0.2 in the univariable analysis were included as covariates, and the outcomes were reported as adjusted hazard ratio (AHR) with 95% CI. The resource use burden for lung involvement in patients with SLE with and without COP was compared using linear logistic regression. All statistical tests were two-sided, with p-values <0.05 deemed to reflect statistically significant associations. Baseline illness severity and mortality risk were assessed and adjusted for using the all-patient refined-diagnosis-related groups (APR-DRG) system, which calculates severity and mortality risk scores at admission. These scores are derived from discharge codes, factoring in primary and secondary diagnoses, age, and preexisting conditions, but excluding codes for complications developed during the hospital stay. The APR-DRG system categorizes patients into four distinct levels of functional loss (LOF) and likelihood of dying (LOD): minor, moderate, major, and extreme, facilitating the assessment and adjustment of illness severity and mortality risk upon admission. The validity of the APR-DRG system has been assessed in a previous study [14]. All analyses were conducted using Stata® software, version 18MP (StataCorp LLC, College Station, TX, USA).

## Results

Missing data

The mean level of covariate missingness in the study was 1.04%, with the “Race” variable having the highest proportion of missing data at 3.07% (Appendix 1). Because of the minimal amount of missing covariate data, a complete analysis of the study cohort was performed.

Incidence and characteristics of COP in SLE hospitalizations

We identified 40,356 adult hospitalizations for SLE during the study period. Of these, there were 3,175 hospitalizations for lung involvement in SLE (79 cases per 1000 hospitalizations for SLE). The mean age of hospitalizations with lung involvement was 44 years (SD, 13.1). The cohort was mostly female (2,822 [88.9%] vs. 352 males [11.1%]), with a higher proportion of Blacks (1,346; 42.4%) than white Americans (937; 29.5%), Hispanics (635; 20%), Asians/Pacific Islanders (127; 4%), or Native Americans (254; 8%).

Among the 3,175 hospitalizations for lung involvement, the most common conditions were interstitial lung disease (488, 15.4%), pulmonary hypertension (206, 6.5%), pleural effusion (650, 20.5%), pleuritis (146, 4.6%), diffuse alveolar hemorrhage (165, 5.2%), nonspecific interstitial pneumonitis (158, 5%), obstructive airway disease (144; 4.5%), bacterial pneumonia (302; 9.5%), acute viral pneumonia (249; 7.7%), and acute respiratory failure (100; 3.1). COP was recorded in 17.9% (570 cases; 14 cases per 1000 adult hospitalizations for SLE, and 180 cases per 1000 hospitalizations for SLE with lung involvement).

Compared with other hospitalizations with other lung involvement in SLE, individuals with COP were significantly older (mean age: 65 vs. 44.3 years; p<0.001), mostly female (515; 90.4% vs. 2,305; 88.5%; p=0.572), had a greater baseline risk of mortality (DRG major or extreme likelihood of dying: 360; 63.1% vs. 1,133; 43.5%; p<0.001), and had a higher prevalence of peripheral vascular disease (25; 4.4% vs. 39; 1.5%; p<0.001), and lower prevalence of lymphocytopenia (45; 7.9% vs. 359; 13.8%; p=0.001), and hypothyroidism (44; 7.8% vs. 357; 13.7%; p=0.001; Table 1).

**Table 1:** Baseline characteristics of lung involvement in SLE with and without COP Table data is presented as totals (N) with percentages (%) except for Mean age. * Significant at values <0.05 LISLE: lung involvement in Systemic Lupus Erythematosus; SLE: Systemic Lupus Erythematosus, COP: cryptogenic organizing pneumonia; SD: standard deviation; CCI: Charlson comorbidity index; DRG: diagnosis-related groups; LOF: levels of functional loss; LOD: likelihood of dying; MI: myocardial infarction; PCI: percutaneous coronary intervention; CABG: coronary artery bypass graft; COPD: chronic obstructive pulmonary disease; PVD: peripheral vascular disease; DM: diabetes mellitus; CHF: congestive heart failure; CKD: chronic kidney disease; AIDS: acquired immunodeficiency syndrome; CREST: calcinosis, Raynaud's phenomenon, esophageal dysmotility, sclerodactyly, and telangiectasia. * Sundararajan's adaptation of the modified Deyo's CCI. This adaptation classifies the CCI into four distinct groups, each indicative of escalating comorbidity and mortality risk. A CCI score surpassing 3 is associated with an approximate 25% 10-year mortality rate, whereas scores of 2 or 1 correspond to 10% and 4% 10-year mortality rates, respectively. ** Median household income quartiles for patient's ZIP Code defined as first quartile = $1 - $49,999; second quartile = $50,000 - $64,999; third quartile = $65,000 - 85,999; and fourth quartile = $86,000 or more.

Variables	Other LISLE (N=2,605), % Unless Otherwise Specified	SLE + COP (N=570), % Unless Otherwise Specified	p-value*
Mean age, years ± SD	44.3 ± 16.8	65 ± 8.1	<0.001
Female	2,305 (88.5)	515 (90.4)	0.572
Insurance status			0.198
Medicare	857 (32.9)	195 (34.2)	-
Medicaid	789 (30.3)	210 (36.9)	
Private	802 (30.8)	154 (27.0)	
Self-pay	159 (6.1)	10 (1.8)	
Race			0.854
White Americans	298 (29.8)	163 (28.6)	-
Black	1,107 (42.5)	239 (42.0)	
Hispanic	511 (19.6)	122 (21.4)	
Asian/Pacific Islander	94 (3.6)	31 (5.4)	
Native Americans	21 (0.8)	5 (0.9)	
Others	99 (3.8)	10 (1.8)	
CCI*			0.322
1	1,019 (39.1)	235 (41.2)	-
2	667 (25.6)	120 (21.1)	
≥ 3	922 (35.4)	215 (37.7)	
DRG severity class			0.101
Minor LOF	250 (9.6)	0 (0.0)	-
Moderate LOF	672 (25.8)	105 (18.4)	
Major LOF	1,115 (42.8)	245 (43.0)	
Extreme LOF	570 (21.9)	220 (38.6)	
DRG risk of mortality class			<0.001
Minor LOD	698 (26.8)	70 (12.3)	-
Moderate LOD	774 (29.7)	140 (24.6)	
Major LOD	826 (31.7)	260 (45.6)	
Extreme LOD	307 (11.8)	100 (17.5)	
Elective admission	65 (2.5)	10 (1.8)	0.656
Admission day is a weekend	584 (22.4)	155 (27.2)	0.285
Median household income (quartile) **			0.974
First (0-25th)	854 (32.8)	192 (33.6)	-
Second (26th– 50th)	610 (23.4)	124 (21.8)	
Third (51st-75^th^)	623 (23.9)	145 (25.5)	
Fourth (76th-100th)	518 (19.9)	109 (19.1)	
Hospital location/teaching status			0.137
Rural hospital	78 (3.0)	40 (7.0)	-
Metropolitan hospital	352 (13.5)	75 (13.2)	
Teaching hospital	2,175 (83.5)	455 (79.8)	
Hospital bed size			0.467
Small	435 (16.7)	110 (19.3)	-
Medium	628 (24.1)	160 (28.1)	
Large	1,542 (59.2)	526 (52.6)	
Hospital region			0.477
Northeast	544 (20.9)	85 (14.9)	-
Midwest	530 (16.5)	110 (19.3)	
South	1,045 (40.1)	254 (44.7)	
West	589 (22.6)	120 (21.1)	
Patient location			0.227
“Central” counties of metro areas of ≥1million population	1,133 (43.5)	239 (42.0)	-
“Fringe” counties of metro areas of ≥1 million population	552 (21.2)	122 (21.4)	
Counties in metro areas with 250,000–999,999 people	565 (21.7)	102 (17.9)	
Counties in metro areas with 50,000–249,999 people	186 (7.2)	31 (5.4)	
Micropolitan counties	145 (4.4)	46 (8.0)	
Not metropolitan or micropolitan counties	52 (2.0)	31 (5.4)	
Comorbidities			
Dyslipidemia	352 (13.5)	125 (21.9)	0.025
Hypertension	690 (26.5)	210 (36.8)	0.030
Smoking	729 (28.0)	150 (26.3)	0.719
Obesity	268 (10.3)	45 (7.9)	0.442
Coronary artery disease	211 (8.1)	60 (10.5)	0.416
Sleep apnea	172 (6.6)	50 (8.8)	0.433
Old MI	96 (3.7)	15 (2.6)	0.585
Old PCI	70 (2.7)	20 (3.5)	0.647
Old CABG	57 (2.2)	5 (0.9)	0.359
COPD	672 (25.8)	150 (26.3)	0.910
Old stroke	83 (3.2)	5 (0.9)	0.176
Dementia	18 (0.7)	5 (0.9)	0.880
PVD	39 (1.5)	25 (4.4)	0.046
Hypothyroidism	357 (13.7)	44 (7.8)	0.001
Hyperthyroidism	13 (0.5)	5 (0.9)	0.631
Lymphocytopenia	359 (13.8)	45 (7.9)	0.001
DM type 1 and 2	313 (12.0)	75 (13.2)	0.744
Obesity	268 (10.3)	45 (7.9)	0.442
CHF	584 (22.4)	140 (24.6)	0.623
CKD	602 (23.1)	145 (25.4)	0.594
Hemiplegia/paraplegia	13 (0.5)	5 (0.8)	0.631
Liver disease	141 (5.4)	20 (3.5)	0.393
Cancer	18 (0.7)	1 (0.1)	0.357
Fibromyalgia	211 (8.1)	55 (9.7)	0.595
Antiphospholipid syndrome	211 (8.1)	20 (3.5)	<0.001
Osteoporosis	89 (3.4)	15 (2.6)	0.668
Hypocomplementemia	13 (0.5)	5 (0.9)	0.630
Major depressive disorder	8 (0.3)	10 (1.8)	0.059
AIDS	8 (0.3)	1 (0.1)	0.596
Lupus nephritis	646 (24.8)	130 (22.8)	0.658
Raynaud’s syndrome	263 (10.1)	45 (7.9)	0.484
CREST syndrome	8 (0.3)	1 (0.1)	0.596
Sjogren’s syndrome	10 (0.4)	5 (0.8)	0.227

Predictors of acute cryptogenic organizing pneumonia

Several factors were associated with an increased likelihood of COP on multivariable regression analysis. These factors include female sex [AHR: 1.46; 95% CI: 1.12-2.96; P=0.022]; hospitalizations occurring in the third quarter of the year (AHR: 1.37; 95% CI: 1.05-2.23; P=0.038); hospital stays of six days or longer (AHR: 1.71; 95% CI: 1.06-2.77; P=0.029); undergoing five or more procedures during the same hospitalization (AHR: 1.56; 95% CI: 1.26-3.56; P=0.041); coexisting lymphocytopenia (AHR: 1.92; 95% CI: 1.16-3.19; P=0.011); need for mechanical ventilation (AHR: 1.60; 95% CI: 1.48-3.93; P=0.049), presence of another autoimmune disorder (AHR: 1.37; 95% CI: 1.15-4.29; P=0.040), and being hospitalized at private, investor-owned hospitals (AHR: 2.62; 95% CI: 1.03-6.64; P=0.043). Conversely, hypothyroidism was associated with a reduced risk of COP (AHR: 0.27; 95% CI: 0.10-0.71; P=0.008; Table 2).

**Table 2:** Univariable and multivariable Cox regression models of predictors of cryptogenic organizing pneumonia in SLE Only covariates with a significance level of P<0.2 were entered into the multivariable logistic regression analysis. p-values <0.05 on multivariable logistic regression were considered statistically significant SLE: systemic lupus erythematosus; HMO: health maintenance organization; LOF: loss of function; DRG: diagnosis-related groups; LOD: likelihood of dying; MI: myocardial infarction; PCI: percutaneous coronary intervention; CABG: coronary artery bypass graft; COPD: chronic obstructive pulmonary disease;  PVD: peripheral vascular disease; DM: diabetes mellitus; CHF: congestive heart failure; CKD: chronic kidney disease; AIDS: acquired immunodeficiency syndrome; DRG: diagnosis-related groups; * Median household income quartiles for the patient's ZIP Code are defined as (First) $1 - $49,999; (Second) $50,000 - $64,999; (Third) $65,000 - 85,999; and (Fourth) $86,000 or more. * Includes Sjogren syndrome, systemic sclerosis, antiphospholipid syndrome, autoimmune thyroiditis, autoimmune hemolytic anemia, idiopathic thrombocytopenia purpura, celiac disease, inflammatory myopathy, or rheumatoid arthritis

Variables	Univariate Logistic Regression		Multivariate Logistic Regression	
	Unadjusted HR	p-value	Adjusted HR	p-value
Female	1.22	0.172	1.46	0.022
Age				
Age ≥ 18 and < 40	1.14	0.528	-	-
Age ≥ 40 and < 60 y	1.02	0.866	-	-
Age ≥ 60 y	1.07	0.524	-	-
Number of procedures				
0-4	0.88	0.564		
≥5	1.92	0.038	1.56	0.041
Discharge quarter				
First quarter	Reference	Reference	Reference	Reference
Second quarter	0.88	0.698	-	-
Third quarter	1.45	0.182	1.37	0.038
Fourth quarter	1.11	0.726	-	-
Hospital control				
Government	Reference	Reference	Reference	Reference
Private, not-for-profit	1.73	0.117	1.37	0.395
Private, investor-owned	2.87	0.021	2.62	0.043
Hospital region				
Northeast	Reference	Reference	Reference	Reference
Midwest	1.64	0.183	1.54	0.290
South	1.56	0.144	1.32	0.466
West	1.30	0.448	1.30	0.523
Insurance status				
Medicare	Reference	Reference	-	-
Medicaid	1.17	0.555	-	-
Private including HMO	0.84	0.532	-	-
Self-pay	0.29	0.099	0.28	0.102
Charlson comorbidity index				
1	Reference	Reference	-	-
2	0.78	0.391	-	-
≥3	1.01	0.966	-	-
Patient location				
“Central” counties of metro areas of ≥1million population	Reference	Reference	-	-
“Fringe” counties of metro areas of ≥1 million population	1.05	0.879	-	-
Counties in metro areas with 250,000–999,999 people	0.85	0.586	-	-
Counties in metro areas with 50,000–249,999 people	0.77	0.597	-	-
Micropolitan counties	1.87	0.157	1.08	0.884
Not metropolitan or micropolitan counties	2.81	0.071	1.31	0.699
DRG severity class				
Minor LOF	Reference	Reference	Reference	Reference
Moderate LOF	0.40	0.002	0.40	0.064
Major LOF	0.57	0.020	0.58	0.104
Extreme LOF	0.99	0.847	-	-
DRG risk of mortality class				
Minor LOD	Reference	Reference	Reference	Reference
Moderate LOD	1.80	0.089	0.88	0.776
Major LOD	3.14	0.001	1.08	0.885
Extreme LOD	3.24	0.002	0.93	0.912
Median household income (quartile)*				
First (0-25th)	Reference	Reference	-	-
Second (26th– 50th)	0.91	0.751	-	-
Third (51st-75^th^)	1.04	0.890	-	-
Fourth (76th-100th)	0.94	0.833	-	-
Hospital location/teaching status				
Rural hospital	Reference	Reference	Reference	Reference
Metropolitan hospital	0.41	0.099	0.47	0.314
Teaching hospital	0.40	0.053	0.57	0.411
Hospital bed size				
Small	Reference	Reference	-	-
Medium	1.01	0.977	-	-
Large	0.77	0.376	-	-
Length of hospital stay				
1-2 days	0.72	0.221	-	-
3-4 days	0.78	0.287	-	-
≥6 days	1.54	0.041	1.71	0.029
Dyslipidemia	1.80	0.027	1.60	0.103
Lymphocytopenia	1.61	0.031	1.92	0.011
Smoking	0.92	0.719	-	-
Obesity	0.74	0.443	-	-
Coronary artery disease	1.33	0.417	-	-
Sleep apnea	1.35	0.434	-	-
Old MI	0.71	0.587	-	-
Old PCI	1.31	0.648	-	-
Old CABG	0.39	0.376	-	-
COPD	1.03	0.910	-	-
Old stroke	0.27	0.207	-	-
Dementia	1.19	0.880	-	-
PVD	3.06	0.068	2.66	0.128
Hypothyroidism	0.54	0.129	0.27	0.008
Hyperthyroidism	1.79	0.635	-	-
Hypertension	1.54	0.129	0.92	0.838
DM type 1 and 2	1.11	0.744	-	-
CHF	1.13	0.623	-	-
CKD	1.14	0.594	-	-
Hemiplegia/paraplegia	1.79	0.635	-	-
Liver disease	0.64	0.397	-	-
Fibromyalgia	1.21	0.596	-	-
Presence of another autoimmune disorder **	I.41	0.051	1.37	0.040
Need for mechanical ventilation	1.67	0.102	1.60	0.049
Osteoporosis	0.76	0.669	-	-
Hypocomplementemia	1.04	0.129	0.89	0.553
Major depressive disorder	7.25	0.106	9.6	0.385
AIDS	0.99	0.921	-	-
Lupus nephritis	0.90	0.658	-	-
Raynaud’s syndrome	0.77	0.485	-	-

Association of COP with outcomes in SLE

Among the 3,175 SLE patients with lung involvement hospitalized between 2016 and 2020, 480 (12.2%) died during the index hospitalization. In the univariate analysis, death in SLE with lung involvement was correlated with age ≥ 60 years [HR (95% CI) 1.16 (1.05-1.56); p=0.01], coexisting lupus nephritis [HR (95% CI), 2.44 (2.04-3.49); p=0.031], cancer [HR (95% CI), 3.49 (2.19-5.79); p<0.001], liver failure [HR (95% CI), 9.82 (4.79-12.57); p<0.001]; immune deficiency [HR (95% CI), 2.22 (2.02-3.11); p=0.031], hypothyroidism [HR (95% CI), 4.67 (1.47-7.750; P=0.009], and high blood pressure [HR (95% CI), 3.15 (2.83-4.51); p<0.001]. In the multivariable analysis, COP remained significantly associated with an increased risk of death [AHR (95% CI), 1.43 (1.16-2.74); p=0.031; Table 3].

**Table 3:** Uni‐ and multivariable Cox regression analyses of risk factors for mortality in SLE with lung involvement Variables with p-values <0.2 in the univariate analysis were added into the multivariable Cox regression; significance level was set at p<0.05 in the multivariate regression analysis HR: hazards ratio; CCI: Charlson comorbidity index; MI: myocardial infarction; LOF: loss of function. **Based on All patient refined-diagnosis illness severity and risk of mortality subclasses * Includes Sjogren syndrome, systemic sclerosis, antiphospholipid syndrome, autoimmune thyroiditis, autoimmune hemolytic anemia, idiopathic thrombocytopenia purpura, celiac disease, inflammatory myopathy, or rheumatoid arthritis

Variables	Univariable Regression		Multivariable Regression	
	Unadjusted HR	p-value	Adjusted HR	p-value
Age ≥ 60 years	1.16	0.012	0.98	0.051
CCI ≥ 3	5.16	0.038	1.97	0.588
Immune deficiency	2.22	0.031	1.91	0.061
Comorbidities				
Coronary artery disease	3.32	0.076	2.14	0.271
Chronic liver disease	9.82	<0.001	5.56	0.002
Hypothyroidism	4.67	0.009	1	-
Hypertension	3.15	<0.001	6.13	<0.001
Old stroke	7.52	0.014	6.71	0.077
Congestive heart failure	3.00	0.053	1.46	0.652
Old MI	5.59	0.034	6.21	0.008
Hypocomplementemia	21.08	0.015	13.5	0.003
Lymphocytopenia	4.67	0.009	1	-
Lupus nephritis	2.44	0.031	1.49	0.054
Cancer	3.49	<0.001	3.01	0.012
Peripheral vascular disease	10.08	0.006	6.49	0.001
Cryptogenic organizing pneumonia	3.84	0.044	1.43	0.031
Coexisting autoimmune disorder*	1.02	0.085	1	-
Extreme LOF at admission**	1.01	0.032	1	-
Major likelihood of mortality at admission**	4.41	0.001	5.92	0.028

Compared with other lung involvement in SLE, COP was not correlated with higher hospitalization costs ($US 94,772 ± 14,759 vs. 95,982 ± 32,625; p=0.954) or longer hospital stay (mean length of hospital stay: 8.3 vs.6.8 days; p=0.147).

## Discussion

Lung involvement in SLE predicts higher mortality and lower quality of life than the general population and SLE without lung involvement [15]. Pulmonary manifestations are prevalent in SLE, with a significant subset of these patients remaining asymptomatic. These manifestations affect every area of the respiratory system and may be the initial presentation of SLE [16]. The necessity for prompt and accurate differential diagnosis in LISLE is predicated upon the critical need for early and accurate diagnosis of the inflammatory process present in the lungs. This urgency stems from the fact that biological therapies, while highly effective for targeted conditions, are designed to modulate or suppress specific pathways of the immune system [17]. Misclassification of pulmonary inflammation in SLE and the consequent use of a biologic agent targeting an incorrect pathway can render treatment ineffective and delay appropriate therapeutic intervention. Such delays exacerbate patient outcomes and elevate the risk of complications. Thus, precise identification of the inflammatory response is critical to the efficacy of biologic therapy for specific lung manifestations in SLE. Such misdirection can delay administering the appropriate therapeutic interventions, thereby escalating the risks for SLE patients [18,19].

The present study identifies a pattern suggesting an elevated likelihood of COP, associated with several factors. These include a higher incidence in females, hospitalizations predominantly occurring in the third quarter of the year (likely due to the summer flare phenomenon or the July effect when new junior doctors are recruited into the workforce), hospital stays extending six days or longer, undergoing five or more medical procedures during a single hospital stay, the coexistence of lymphocytopenia, the necessity for mechanical ventilation, the presence of another autoimmune disorder, and hospitalization in private or investor-owned facilities. Conversely, the study identified a diminished association between hypothyroidism and the risk of developing COP.

The observed higher incidence of COP among females may be linked to the higher hospitalization for SLE among women compared with men [20], a phenomenon whose underlying causes remain to be fully elucidated. Previous studies have suggested that this gender predisposition could be attributed to differences in the metabolism of sex hormones and/or gonadotropin-releasing hormone (GnRH) [21]. Furthermore, the increased flare-ups of SLE symptoms during the summer months, particularly in the third quarter of the year, have been well documented and were corroborated by our study's findings. Environmental factors, particularly sunlight and ultraviolet rays, are significant in triggering SLE symptoms. Consequently, a surge in SLE hospitalization is logically expected in the summer, characterized by stronger sunlight, more potent UV radiation exposure, and accumulation of photosensitivity. Additionally, the extended hospital stays observed among patients can be attributed to the severity of their condition. Hence, it can be inferred that patients with SLE and concurrent COP, being more severely affected, tend to have longer hospitalizations.

Lymphocytopenia has been identified as a marker of increased disease activity in SLE, and with heightened disease activity, the likelihood of pulmonary involvement, including COP, escalates [22]. Lymphopenia at presentation has been associated with an increased risk of systemic infections in SLE [23]. SLE can present as COP, independent of the disease's activity level [4,24].

Additionally, the use of immunosuppressive treatments in managing lupus heightens the risk of respiratory infections, which may present symptoms similar to acute pulmonary manifestations of SLE. Despite some patients being asymptomatic, with respiratory disorders only detected through imaging or pulmonary function tests, the complexity of these conditions necessitates treatment decisions often guided by case reports or small series, due to the limited clinical trial data specifically addressing pulmonary manifestations of SLE. Therapeutic strategies frequently draw on evidence from severe SLE cases affecting other organs or from the treatment of pulmonary manifestations in other systemic autoimmune rheumatic diseases [25-27].

Few studies have examined the impact of COP on mortality rates and other outcomes in SLE or its contribution to the burden of pulmonary manifestations in SLE. Pulmonary involvement is common in systemic lupus erythematosus (SLE), affecting 50% to 70% of patients and serving as the initial presenting feature in 4% to 5% of cases. Within ten years of diagnosis, approximately 12% of patients develop permanent lung damage [27]. Pulmonary vasculitis, also known as diffuse alveolar hemorrhage, is a rare yet severe complication of SLE that carries a high mortality rate reported to reach up to 90% [28,29]. Recent studies have also associated pneumonia, respiratory failure, extensive pulmonary fibrosis, and need for endotracheal intubation with increased 30-days mortality in SLE patients [30,31]. In addition to age ≥ 60 years, coexisting lupus nephritis, cancer, liver disease, immune deficiency, hypothyroidism, and high blood pressure, COP was significantly associated with a higher risk of death in the index study. Although COP generally responds well to corticosteroids with clinical-radiographic resolution in 70-80% of cases [32], it has been reported to rapidly worsen and lead to respiratory failure that is refractory to corticosteroids [33]. Contributing factors to mortality include delayed diagnosis or treatment, severe initial presentation, coexisting conditions such as autoimmune diseases, increased risk of infections due to immunosuppressive therapy, complications from long-term steroid use, resistance to standard treatments, and progression to pulmonary fibrosis. These elements can complicate COP management, exacerbating the condition and increasing the mortality risk [34].

The concurrent use of immunosuppressants and steroids in SLE patients poses challenges including managing a broad spectrum of side effects (such as increased infection risk, osteoporosis, weight gain, hypertension, diabetes, and mood swings), steroid dependency and resistance, flare management, adverse impacts on bone health, elevated cardiovascular and metabolic risks, and adrenal suppression [35]. These factors necessitate a careful, individualized approach to balance the benefits of controlling SLE activity with the potential risks associated with long-term steroid therapy, requiring vigilant monitoring and management strategies to mitigate adverse outcomes. Various etiologic agents suggested to increase the risk of COP have been associated with SLE flare-ups including viral infections [36], environmental factors and toxicants [37], gastro-esophageal reflux [38], connective tissue disorders [39], and medications often used in SLE patients such as methotrexate and cyclophosphamide [40].

Limitations

The Nationwide Inpatient Sample (NIS) captures data on hospitalizations rather than individual patients, making it challenging to identify repeat admissions for the same individuals. The retrospective design of the study restricts the ability to establish causality, allowing only for the identification of associations. Additionally, the NIS lacks information on factors such as medication adherence, patient lifestyle, and outpatient treatments for mild lung involvement in SLE, all of which may influence hospitalization rates and outcomes for patients with cryptogenic organizing pneumonia. Future studies examining these factors will enhance our understanding of the burden and contributors to COP in SLE. While the NIS offers a comprehensive and diverse dataset, its scope is confined to U.S. healthcare facilities, limiting the generalizability of the findings to other populations. Diagnosing COP, a diagnosis of exclusion, typically requires lung biopsy or radiological imaging to rule out other causes of pneumonia. This study does not address the mortality risks associated with biopsy procedures or report the number of patients who required mechanical ventilation after bronchoscopy. Moreover, it does not explore the impact of diagnostic delays on mortality or the potential effect of prolonged hospital stays due to diagnostic procedures on mortality outcomes. Despite these limitations, the study effectively highlights the burden of COP in SLE patients, demonstrating its impact on clinical outcomes and resource utilization during SLE-related hospitalizations.

## Conclusions

Approximately 8% of hospitalizations for SLE involve pulmonary complications, with 1% of these cases being further complicated by COP. Factors such as female sex, extended duration of hospital stay, admissions during the third quarter of the year, undergoing multiple hospital procedures or mechanical ventilation, and a significant comorbidity burden substantially increased the risk of COP. COP is associated with an elevated risk of mortality among hospitalized SLE patients. Given the efficacy of steroids in the management of COP, further investigation into early detection methods and intervention strategies, the refinement of steroid therapy to enhance benefits and mitigate side effects, and the clarification of the contribution of identified risk factors of COP, will significantly benefit SLE management. Moreover, it is essential to improve management strategies for comorbidities, address issues of medication non-adherence, and update guidelines for alternative or adjunctive therapies, including immunosuppressants and biologics, especially in cases of steroid-resistant COP or when reducing steroid-induced adverse effects is paramount.

## Appendices

Appendix 1 

**Table 4:** Missing cohort data

Variable	Missing	Total cohort	Percent Missing
Died in the hospital	6	3175	0.19
Discharge disposition from the hospital	6	3175	0.19
Elective vs. nonelective admission	6	3175	0.19
Patient location	24	3175	0.77
Race	97	3175	3.07
Total hospital charges	37	3175	1.15
Transfer from other hospitals	6	3175	0.19
Transfer to other hospitals	6	3175	0.19
Median annual income quartile in the patient's ZIP code	55	3175	1.73
Insurance status	85	3175	2.69
